# A critical analysis of Lin et al.'s (2021) failure to observe forward entrainment in pitch discrimination

**DOI:** 10.1111/ejn.15778

**Published:** 2022-08-03

**Authors:** Kourosh Saberi, Gregory Hickok

**Affiliations:** ^1^ Department of Cognitive Sciences University of California Irvine California USA; ^2^ Department of Language Science University of California Irvine California USA

**Keywords:** attention, auditory, periodicity, phase, psychophysics

## Abstract

Forward entrainment refers to that part of the entrainment process that outlasts the entraining stimulus. Several studies have demonstrated psychophysical forward entrainment in a pitch‐discrimination task. In a recent paper, Lin et al. (2021) challenged these findings by demonstrating that a sequence of 4 entraining pure tones does not affect the ability to determine whether a frequency modulated pulse, presented after termination of the entraining sequence, has swept up or down in frequency. They concluded that rhythmic sequences do not facilitate pitch discrimination. Here, we describe several methodological and stimulus design flaws in Lin et al.'s study that may explain their failure to observe forward entrainment in pitch discrimination.

## Graphical abstract

Several studies have demonstrated psychophysical forward entrainment in a pitch‐discrimination task. In a recent paper, Lin et al. (2021) challenged these findings by demonstrating that a sequence of 4 entraining pure tones does not affect the ability to determine whether a frequency modulated (FM) pulse, presented after termination of the entraining sequence, has swept up or down in frequency. Here, we describe several methodological and stimulus design confounds in Lin et al.'s study that may explain their failure to observe forward entrainment in pitch discrimination.

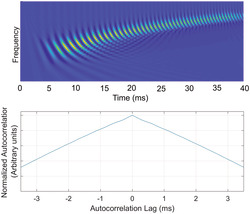

**Abbreviations**
CF: centre frequency
FM: frequency modulation

## 1 INTRODUCTION

Forward entrainment refers to that part of neural or psychophysical entrainment that outlasts the entraining stimulus (Saberi & Hickok, 2021). In an influential study, Jones et al. (2002) demonstrated forward entrainment in pitch discrimination driven by the temporal expectancy set by an entraining sequence of 9 tones (a standard tone followed by 8 more entraining tones). After the final tone in the driving sequence, a comparison tone was presented that either had the same frequency (pitch) as the standard or was higher or lower by one semitone. The subject's task was to indicate whether the pitch of the comparison tone was higher, lower or the same as the standard. The critical variable was the comparison tone's onset time, which was set either at the expected temporal interval or slightly off. They found that pitch discrimination accuracy modulated as a function of the comparison tone's temporal position, with best performance observed when it was presented at the expected time set by the terminated entraining sequence. Performance declined proportionally with the degree of deviation from the expected position. They further showed that this discrimination pattern appears to be periodic, with performance once again improving as deviation from expected time approached *twice* the expected temporal interval. They speculated that this effect is based on a purely reflexive adaptive shift of attention in time towards the temporal locus of the target sound. Jones and colleagues confirmed these findings in several follow‐up studies (Barnes & Johnston, 2010; Barnes & Jones, 2000; Ellis & Jones, 2010; Jones et al., 2006).

In a recent paper, Lin et al. (2021) reported the results of experiments in which rhythmic sequences were used to cue the discrimination of 40‐ms frequency modulated (FM) pulses that either swept up or down in frequency. Specifically, they aimed to test the predictions that (1) a rhythmic cue will improve discrimination of the pitch of a target that occurs in phase with the rhythmic cue, (2) that this behavioural benefit will persist for a number of cycles after the external rhythm stops and (3) the behavioural benefit of entrainment would be strongest for rhythmic rates closest to that of endogenous oscillations (Haegens & Golumbic, 2018; Obleser & Kayser, 2019). In their study, Lin et al. (2021) measured two performance variables, accuracy (proportion correct) and reaction time. They found no forward entrainment in ‘pitch discrimination’ contrary to findings of Jones et al. (2002) and, more broadly, no evidence in support of any of the three predictions described above. In this paper, we evaluate several methodological and stimulus design flaws in Lin et al.'s study that raise questions as to the validity of their findings.

## 2 LIN ET AL.'S MAIN FINDINGS

Lin et al. (2021) describe four experimental conditions comprising two cuing and two target conditions (2 × 2 design). The cue was either a rhythmic sequence of four pure tones (square wave, i.e., 50% duty cycle) or a continuous pure‐tone whose duration matched the total duration of the 4‐tone rhythmic sequence (including intertone intervals). The continuous pure‐tone cue had a fixed duration within a block of trials. Lin et al. inaccurately refer to this steady state and deterministic stimulus as the ‘random‐cue’ condition (see below). Each cue (rhythmic or steady state) was then followed by the target FM pulse. The listener's task was to determine if the FM target swept up or down in frequency. The target was either presented in‐phase with the rhythmic sequence (at the onset of one of the first 4 potential cycles after the end of the rhythmic cue) or at a completely random time after the termination of the cue. The rhythmic sequences had rates of 1.4, 1.7 or 2 Hz in experiment 1 (ISIs of 700, 600 and 500 ms, respectively); 1.1, 1.7 or 2.5 Hz in experiment 2; and ranging from 0.8 to 10 Hz in their third experiment. In the first two experiments, the sequence rate was fixed within a run. In the third experiment, it was randomly selected on each trial from a closed set. The target, a *single* FM pulse, was referred to by the authors as rhythmic when it occurred at one of four prespecified onset times whether or not the cue was rhythmic or continuous. Cue or target types were not mixed within a run, with one exception. On a small subset of trials (20% of ‘rhythmic cue’‐‘rhythmic target’ condition), they presented targets that were antiphasic to the rhythmic cue. The main (and most relevant) finding from Lin et al. (2021) was the absence of a selective advantage of a rhythmic cue in any of the tested conditions (no forward entrainment). That is, the rhythmic cue did not improve performance for the ‘rhythmic’ target relative to the random target, nor did the rhythmic cue provide a selective advantage compared to the steady state cue for the ‘rhythmic’ target.

## 3 EXPERIMENTAL DESIGN FLAWS AND CONFOUNDS

The methodological concerns we have with this study are as follows. First, for rhythmic targets, they averaged performance across 4 cycles after termination of the cue in nearly all of their analyses. This clearly dilutes any potential forward entrainment effect, which is strongest for the first poststimulus cycle, and absent by the 3rd or 4th cycles as others studies have shown (Forseth et al., 2020; Hickok et al., 2015). It is unclear why Lin et al. averaged potential forward entrainment effects across 4 cycles when Jones et al. (2002) have shown the effect only for the first two cycles, and others have explicitly shown that there is no forward entrainment effects at Cycles 3 and 4.

The peer‐review history of Lin et al.'s paper (https://publons.com/publon/10.1111/ejn.15208) shows that both reviewers raised concerns about the effects of this averaging procedure on diluting entrainment effects. The authors, however, chose to maintain this approach, adding a brief paragraph in which they report mixed results for the first post‐stimulus cycle with a significant interaction effect between cue and target rhythmicity on accuracy (i.e., an entrainment effect) and several null results. There is no information provided about this latter analysis, no figures and no experimental details. It is unclear, for example, if performance for the rhythmic target at Position 1 was contrasted to the averaged *full* window of the random‐target condition. In this likely scenario, potential entrainment effects will be diluted because, as inferred from their hazard‐rate functions, accuracy (in the random‐target case) improves near the end of the target‐presentation window.

Second, the entraining sequence used by Lin et al. comprised only 4 cycles, a significantly smaller number than the 9 cycles used by Jones et al. (2002), Hickok et al. (2015) and Farahbod et al. (2020), as well as a number of others who employed longer sequences (as many as 12 cycles) in demonstrating forward entrainment in a variety of auditory tasks (Barnes & Jones, 2000; Lange, 2009; Lawrance et al., 2014; Rimmele et al., 2011). In fact, Wilsch et al. (2020) in a crossmodal study that used entraining auditory stimuli identical to those used by Lin et al. suggest that ‘perhaps, four cycles is not enough to properly drive the system’.

Forward entrainment has been demonstrated with as few as 4 cycles in vision research (Breska & Deouell, 2017; de Graaf et al., 2013). However, there are differences in entrainment effects across modalities and findings in vision cannot be unambiguously extended to audition. For example, Wilsch et al. (2020) demonstrated both behavioural and neurophysiological differences between how a 4‐cycle visual entraining stimulus affects an auditory target when compared with how a 4‐cycle auditory entraining stimulus affects a visual target. One of their findings, for instance, was that a 4‐cycle visual entraining stimulus affected intertrial phase coherence more significantly in rhythmic trials than random trials, whereas a 4‐cycle auditory entraining stimulus did not (their Figure 5a). Thus, while there may be some crossmodal commonalities in entrainment processes, there are also important differences that caution against drawing parallels without direct evidence. Table 1 shows a summary of several of the more well known *auditory* studies of forward entrainment. With the exception of the Lin et al. and Wilsch et al. studies, most others have used larger numbers of entraining cycles (averaging around 9 cycles). To our knowledge, there are no studies in hearing research that have used 4 (or fewer) cycles of an auditory stimulus to successfully demonstrate forward entrainment of an auditory target.

**TABLE 1:** The number of auditory entraining cycles used by several studies of forward entrainment

Study	# of entraining stimuli
Lawrance et al. (2014)	7
Hickok et al. (2015)	9
Farahbod et al. (2020)	9
Barnes and Jones (2000)	9
Jones et al. (2002)	9
Lange (2009)	12
Ellis and Jones (2010)	6
Rimmele et al. (2011)	12
Sanabria and Correa (2013)	6
Simon and Wallace (2017)	9
Forseth et al. (2020)	9
Bauer et al. (2015)	9
Sun et al. (2021)	9
Lin et al. (2021)	4
Wilsch et al. (2020)	4

Although the time course of entrainment as a function of the number of entraining cycles has not been systematically studied, there is one study of which we are aware that definitively shows a build‐up in the temporal dynamics of neural entrainment (Bauer et al., 2018). This study, however, is related to simultaneous (not forward) entrainment, and it is unclear how the temporal evolution of neural entrainment affects performance after termination of the entraining stimulus. Currently, what we can state definitively is that all studies of auditory forward entrainment that have shown a positive (modulatory) effect on performance have used a larger number of entraining cycles than that used by Lin et al., and this should be noted as a factor in evaluating their null results.

One final clarification worth making about the number of entraining cycles is that a single isolated cue (1 cycle) can in fact reset the phase of an endogenous oscillator. Stefanics et al. (2010), for example, have shown that the phase of an electroencephalographic (EEG) delta rhythm can be reset with a single auditory cue with concomitant cyclic effects on behavioural reaction times. The effect was shown to be greatest when the informational content of the cue was more predictive of whether or not the next tone (after the cue) was a target tone (a higher frequency tone indicated to the subject that it is more likely that the next tone, occurring either 1350 or 2700 ms later, would be a target tone). However, this study and similar single‐cue studies with long cue‐target delays generate expectations based primarily on informational or symbolic cuing (Posner, 1980; Treisman, 1963) and not on use of implicit cues that capture attention or other involuntary rhythmic‐coding (automatic) processes. Furthermore, by definition, a single cue does not generate a periodic expectation as is the case for the sequence of entraining stimuli used by Jones et al. (2002), Lin et al. (2021) and other studies of forward entrainment. Stefanics' paradigm of using a single stimulus to reset the phase of an endogenous oscillator, while a valuable contribution to understanding the nature of neural oscillations, is in our opinion a categorically different phenomenon, one that is inconsistent with the definition of entrainment advanced by other research groups. Lakatos et al. (2019), for example, propose restricting the definition to a particular type of neural phenomenon in which a rhythmic stimulus directionally and repetitively resets the phase of an endogenous neural oscillator, distinguishing between this phenomenon and transient phase resetting resulting from a single external event (e.g., Stefanics) and bidirectional effects in coupled oscillators (see Saberi & Hickok, 2021).

**FIGURE 1:**
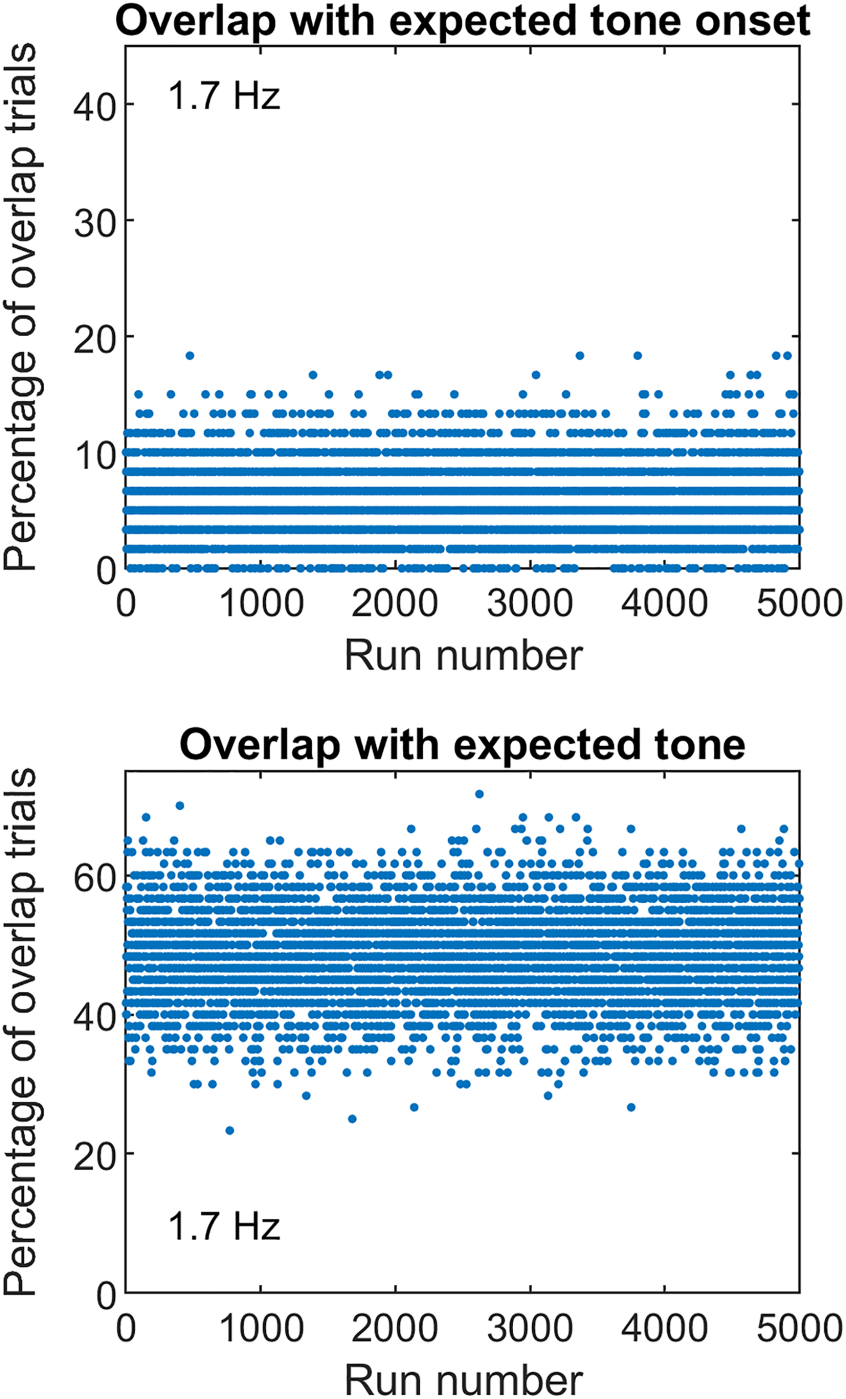
Monte Carlo simulations showing the percentage of trials on which the ‘random’ target occurred during the expected (not random) target window in Lin et al. (2021). This confound diluted the difference in forward entrainment between rhythmic and ‘random’ targets. Top panel shows results for overlap with the onset of the temporal expectancy window and bottom for its full duration.

A third major experimental design flaw in the Lin et al. study is that ‘random targets’ could have occurred at *any* time after the end of the cuing sequence. This means that, on a subset of trials, the ‘random targets’ occurred at or very near the ‘in‐phase’ temporal positions. This would have additionally diluted any selective advantage of the in‐phase only condition (rhythmic target) because on an unknown proportion of trials, the ‘random’ target would have overlapped with the expected (cued) position of the in‐phase targets. For the 40‐ms target pulses used by Lin et al., Monte Carlo simulations show that this overlap occurs on a significant number of ‘random target’ trials (Figure 1; 5000 runs). For a 1.7‐Hz rhythmic sequence (600‐ms ISI), the condition closest to that used by Jones et al. (2002), simulations show that on 6% of the ‘random target’ trials (on average), a segment of the FM target overlapped with the onset of the expected tone pulses (had they continued). On some runs, this number could be as high as 15% of trials within a run (top panel). If, however, we assume that the temporal expectancy window generated by the rhythmic cuing sequence is set by the full ‘on period’ (instead of the onset) of the cuing tones, then this overlap occurs on approximately half the trials of a run (bottom panel). These overlap proportions are even larger for the 2‐ and 2.5‐Hz rhythmic sequences. In other words, on a significant number of trials, the ‘random’ FM target could be heard during some portion of the temporal expectancy window generated by the rhythmic sequence, diminishing performance differences between the rhythmic and ‘random’ target conditions. This problem did not exist in the design used by Jones et al. (2002), Hickok et al. (2015) and other studies of forward entrainment because the temporal positions of targets in those studies were restricted to a closed (discrete) set and performance was reported separately for each of those temporal positions.

Fourth, Lin et al. inaccurately refer to use of a deterministic cue (a continuous pure tone) as a ‘random cue’ condition. This cue clearly marked the start time of the rhythmic target in a block design in which target timing was fixed. The authors acknowledge that ‘while “continuous cue” might be the label that better reflects the nature of the cue, we have chosen “random” such that we would have the same labels for the factors *cue* and *target* rhythmicity in this 2x2 design’. The concern, however, is not simply related to inaccurate terminology but rather that use of a deterministic cue diminishes entrainment effects relative to a truly random cue. In a block design, subjects can pick up on the timing of the rhythmic target and use the offset cue as a marker to predict rhythmic target timing which is fixed and repeated on *every* trial. Even the authors concede that the mislabeled ‘random’ cue (i.e., continuous deterministic tone) ‘provides implicit temporal information, and cue offset can be used to predict the timing of the implicit rhythm … if the participant has learned the rhythmic target‐structure over the course of a block’.

Fifth, Lin et al. also averaged data across the 3 antiphasic cycles (1.5, 2.5, 3.5) similar to the in‐phase averaging confound described above and skipped the 0.5 cycle where one would expect the strongest phase effect (near the start of the target observation window). This further stacks the deck against the possibility of detecting forward entrainment. Perhaps one reason why Lin et al. averaged performance across so many conditions, that is, across cue or target types, across target temporal positions and across modulation (entrainment) rates, may have to do with the very small number of trials associated with each target position. For the rhythmic (in phase) condition, each subject completed only 12 trials at each temporal position and rate. This is unusually small for psychophysical research. The problem is even worse for the antiphasic condition where each subject (remarkably) completed only 4 trials at each of the three antiphasic temporal positions. The consequence of this low number of trials is the significant within‐subject variability observed even when data are averaged across conditions. Take for example the individual subject data shown in the top‐right panel of their Figure 2 that compares the effects of cue type on accuracy. Though the authors state that there was no effect of cue type, one subject showed ~95% accuracy (near perfect) in the rhythmic‐cue condition and ~55% accuracy (near chance) in the ‘random’‐cue condition. Many of their participants show similar patterns of large within‐subject variability. 1

Perhaps Lin et al. averaging of performance across cycles was, as suggested to us by one reviewer, partially motivated by their prediction that entrainment should last for ‘a number of cycles after the external rhythm stops’. However, the phrase ‘a number of cycles’ is vague, and the selection of 4 cycles is arbitrary (e.g., if they had averaged across the first two [instead of four] cycles and had found a modulatory pattern of performance, as Jones et al., 2002, Hickok et al., 2015, Farahbod et al., 2020 and others have reported, would that not qualify as entrainment?). Furthermore, why then did they not specifically evaluate whether modulation in performance would be observed at each of four expected cycles (instead of averaging) to determine the time course of entrainment (i.e., how it temporally evolves) as Bauer et al. (2018) have reported. Moreover, if the argument is that they did not have a sufficiently large number of trials at each cycle to analyse performance at individual cycles (with which we agree), then why did they not simply collect more data? This justification seems untenable.

## 4 STRENGTH OF PITCH CUES

Sixth, and most important, the stimuli used by Lin et al. (2021) are not suitable for testing pitch discrimination as has been claimed. They used single 40‐ms FM sweeps whose direction was to be determined by subjects in a single‐interval forced‐choice task. This stimulus is markedly different than that used for pitch discrimination by Jones et al. (2002), which comprised a *pair* of 150‐ms sequentially presented pure tones (standard and comparison). Subjects in Lin et al. were, by our analysis (see below), likely discriminating timbre differences, the perceptual properties of which were learned across trials. Timbre, which is defined by a sound's subjective quality of ‘coloration’ and ‘texture’, requires use of high‐level and computationally more complex processes compared with those involved in simple pitch discrimination (Berger, 1964; Caclin et al., 2005; Grey, 1977; Iverson & Krumhansl, 1993; Jenkins, 1961; Mörchen et al., 2006). Two sounds that have the exact same pitch and loudness could have significantly different timbres (e.g., same musical note played at the same amplitude on two different violins). Models of timbre encoding (Caclin et al., 2005; Grey, 1977; Plomp, 1970) employ multidimensional scaling in dissimilarity space to quantify the timbre of complex sounds. Such high signal dimensionality may not be affected by a brief 4‐cycle entraining stimulus in the same manner (or with the same ease) as lower‐level processes like pitch encoding.

### 4.1 Autocorrelation analysis of pitch cues

Why do we suggest that discrimination performance in Lin et al. is based on timbre cues? There are two ways to think about an FM sound. First, in terms of its instantaneous frequency at any given point in time (this is what Lin et al. assume) and second, in terms of its long‐term spectrum (Hsieh et al., 2012; Rabiner & Gold, 1975; Saberi, 1998; Saberi & Hafter, 1995). The latter interpretation is important given that the auditory system requires a minimum integration window (the system's time constant) to process an acoustic waveform. Although, the long‐term amplitude spectrum of linear up and down sweeps is identical, their phase spectra, which significantly affect timbre (Plomp & Steeneken, 1969), are markedly different (Figure S1). These spectral compositions are further altered differentially between up and down sweeps after passing through auditory filters, adding to their perceptual differences. To gain better insight into the signal processing dynamics and available ‘pitch’ cues that contributed to performance in the Lin et al. study, we processed their stimuli (as well as those of Jones et al., 2002) through an autocorrelation model of pitch extraction (Balaguer‐Ballester et al., 2008; Hsieh & Saberi, 2007; Licklider, 1951; Rabiner, 1977; Shimamura & Kobayashi, 2001). The model comprised a GammaTone filterbank (Holdsworth et al., 1988; Slaney, 1998) with 50 filters whose centre frequencies (CFs) were logarithmically spaced from 300 to ~3000 Hz, followed by an inner hair‐cell model (Meddis et al., 1990; Slaney, 1998) and an autocorrelation function:

(1) $$R\left(t,\tau \right)=\int_{f_{l}}^{f_{u}}\int_{T=0}^{\infty }x\left(f,t-T\right)x\left(f,t-\tau -T\right)e^{-T/0.005}\mathrm{dT}\;\mathrm{df}\;$$

where *x* is the time waveform at the output of each frequency channel, *f*_*l*_ and *f*_*u*_ are the lower and upper filter CFs over which the autocorrelation patterns are integrated, *T* is time into the past relative to current time, the exponential decay has a time constant of 5 ms and τ is the autocorrelation lag.

**FIGURE 2:**
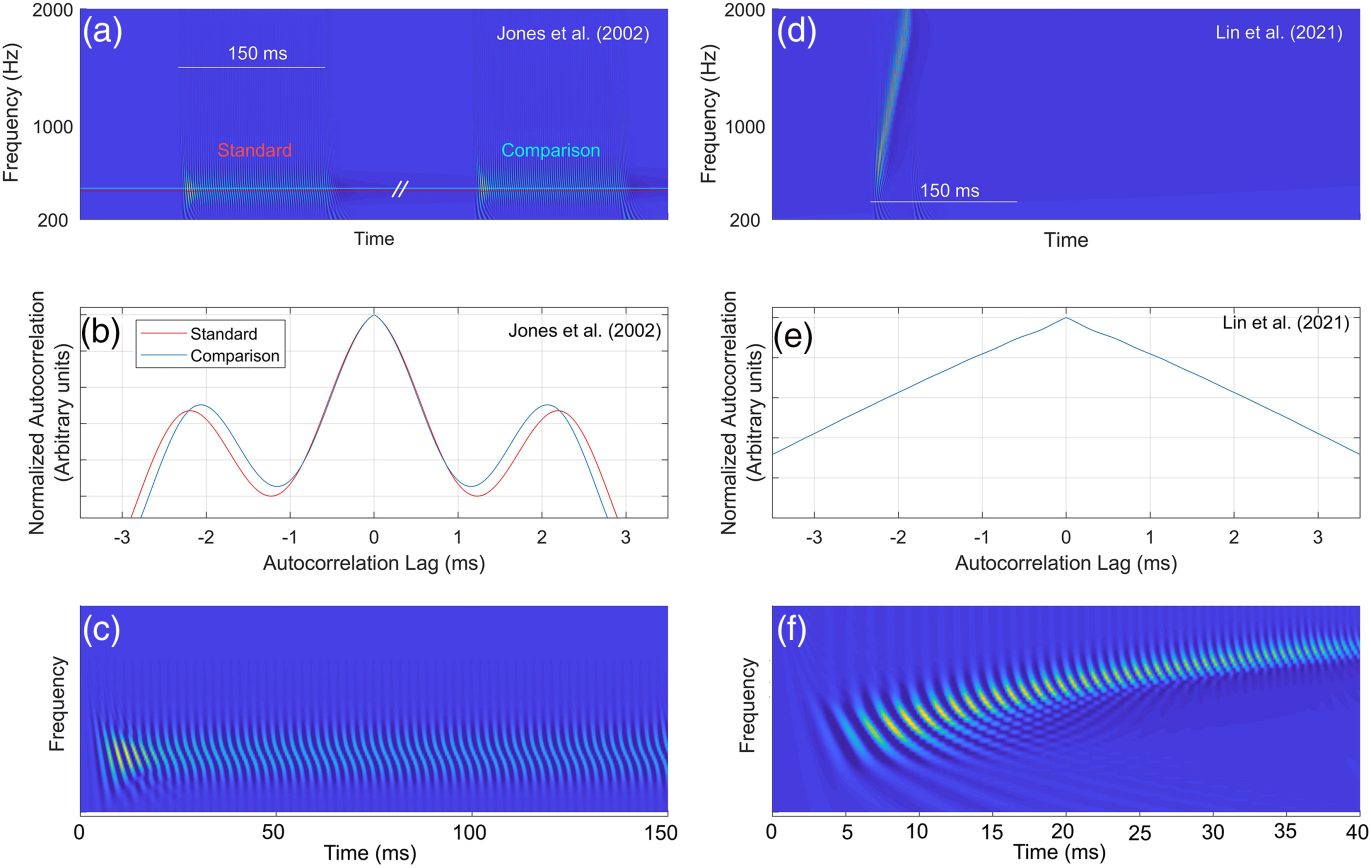
Output of an autocorrelation model of pitch extraction for the type of stimuli used by Jones et al. (2002) (left panels) and Lin et al. (2021) (right panels). No pitch estimate (secondary peak) is obtained from processing Lin et al.'s stimuli through the model. See text for details.

The left panels of Figure 2 show the output of this model in response to sample stimuli used by Jones et al. (2002). This included a standard tone followed, after an ISI, by a comparison tone that is one semitone higher in frequency than the standard tone. 2 Panel (a) shows the time‐by‐frequency output of the filterbank stage. The red and light blue horizontal lines are centred at the frequencies of the standard and comparison tones, respectively. Panel (b) shows the model's normalized autocorrelation function integrated across frequency channels (Equation 1). Peaks at non‐zero lags (near 2 ms) are the model's pitch estimates (i.e., inverse of the lag value). The model clearly predicts significant pitch differences between the two stimuli. Panel (c) shows the model's time‐frequency response to the standard stimulus (magnification of the left trace from panel (a)). The right panels of Figure 2 (panels d–f) show the model's output in response to a 40‐ms FM pulse. In Lin et al.'s (2021) single‐interval task, subjects had to detect whether the FM sweep was up or down (discriminate pitch differences within the single 40‐ms sound). Panel (e) shows that the autocorrelation function produces no pitch estimate for these pulses (no peak at a non‐zero lag). To more carefully analyse the dynamic nature of pitch cues in their stimuli, we used a short‐term (running) autocorrelation function with a 5‐ms exponential decay time constant to dynamically update pitch estimates throughout the duration of the stimulus and again found no pitch estimate (secondary peak) as shown in Video S1. 3

**FIGURE 3:**
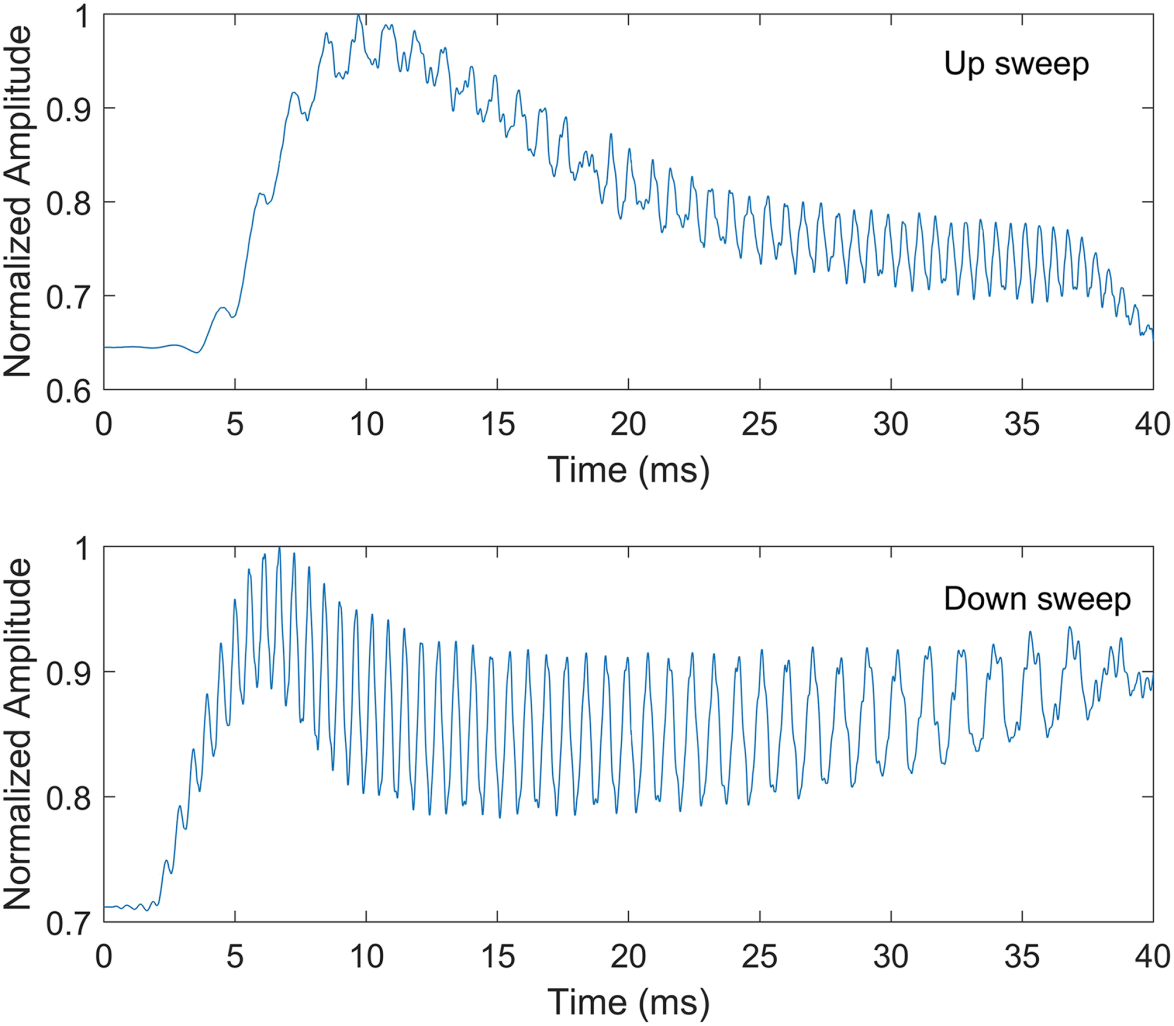
Temporally asymmetric response of an auditory filter model to up and down sweeps. This envelope and fine structure asymmetry contributes to dynamic timbre and loudness differences.

In addition, the response of auditory filters to brief FM pulses is asymmetric, partly because cochlear filters have a sharper slope above the filter's CF and partly due to the nonlinear dynamics of cochlear hair cells. This asymmetry may be observed in Figure 3, which shows the temporal envelope and fine structure response of a cochlear filter model described earlier to the type of 40‐ms FM sweeps used by Lin et al. (2021). Note the asymmetries across the two sweep types in their declining envelope amplitudes as a function of time, as well as the changing AC amplitude (fine structure variance), which is the main determinant of spectral content. For example, in the top panel, the higher frequency segment of the FM will generate greater spectral energy (near 35 ms) than the lower‐frequency part of the FM (near 10 ms) in spite of the larger envelope amplitude at the low‐frequency end. Thus, these stimuli can generate perceptually changing properties, which include dynamic timbre (Iverson & Krumhansl, 1993) and loudness cues. There is also evidence that a greater number of cortical neurons code for up (compared with down) FM sweeps in some regions of the auditory cortex (mediolateral belt) and in the opposite direction in other regions (anterolateral belt) (Tian & Rauschecker, 2004). These neural population differences may potentially further contribute to perceived differences in unknown ways between the two types of pulses and may also be the source of the asymmetry observed in psychophysical performance between identification of up and down FM sweeps (Luo et al., 2007). To be clear, although timbre is by our estimation the predominant cue used to discriminate the up from down FM pulses used by Lin et al., we do not say that there is *no* pitch cue but rather that any such cue would be very weak and potentially confounded with dynamic timbre and loudness cues from asymmetric envelopes at the output of cochlear filters. One can, in fact, force a weak pitch cue at the model's output by introducing a nonlinearity (square or cubic) that enhances the ridges of the filtered FM pulse, though even in this case, such putative cues are significantly weaker than those in the stimuli used by Jones et al. (2002).

Finally, one can also demonstrate that the cues used by subjects in the Lin et al. study are not pitch based either by shifting the entire FM sweep to regions of the acoustic spectrum above 5 kHz (Audio Demo S1: up followed by down sweep, 5.5–7 kHz) where pitch cues as defined by ANSI (1973) are nonexistent (or extremely weak) due to the upper bound on neural phase locking (Ward, 1954; Moore, 1973; Palmer & Russell, 1986; Semal & Demany, 1990; Yost, 2009) or by reducing the duration of the FM sweep to 10 ms (5‐ms rise‐decay; Audio Demo S2: up followed by down sweep, 0.5–2 kHz), too brief to allow salient pitch cue discrimination *within* the pulse. In both cases (shifting above 5 kHz or reducing duration to 10 ms), the same qualitative perceptual differences (largely dynamic timbre and loudness differences) are heard between up/down sweeps as those heard when listening to the sweeps of the type used by Lin et al., that is, 40‐ms FMs at lower frequency regions (Audio Demo S3: 0.5–2 kHz or Audio Demo S4: 0.6–0.9 kHz), with near perfect discriminability of the two types of pulses (up vs. down sweep).

To summarize, the use of FM pulses by Lin et al. is not suitable for detecting forward entrainment in pitch discrimination. Timbre cues likely dominate perception of these pulses. Timbre is complex, multidimensional and a nonmonotonic function of frequency, the discrimination of which requires learning complex perceptual features (e.g., coloration or texture) *across* trials within a run. This is not the case for pure‐tone pitch cues such as those used by Jones et al. (2002). Pitch is a monotonic function of frequency and its discrimination (high vs. low) does not require learning. Furthermore, unlike most studies of forward entrainment that involve *detection* of a stationary signal (e.g., a pure tone), Lin et al. require *discrimination* of a changing perceptual property *within* a single brief pulse (a more demanding task). Entrainment may not as easily enhance discrimination of complex and dynamic perceptual features within brief pulses. Moreover, the entraining stimulus was of a categorically different type (pure tones) compared to the target (FM). A more suitable entraining stimulus, in our opinion, would have been one that cued a within‐stimulus‐class feature, for example, by using an entraining sequence of upsweep FM pulses (or a sinusoidal FM) rather than a sequence of fixed‐frequency pure tones. Finally, there are asymmetries in the population of neurons that code for up vs. down FM sweeps, and psychophysical studies have additionally demonstrated threshold asymmetries in identification of sweep direction (Luo et al., 2007). Such neural population asymmetries, which may contribute in unknown ways to perceptual differences, are not known to exist when discriminating temporally separated pure tones such as those used by Jones et al. (2002) in demonstrating forward entrainment in pitch discrimination.

## 5 CONCLUSION

There are several methodological concerns that raise questions as to the validity of findings reported by Lin et al. (2021). These include averaging performance across 4 poststimulus cycles, use of short entraining sequences (4 cycles), confounds associated with use of deterministic (instead of truly random) cues in a block design that provided a predictive marker to target timing, overlap between ‘random’ target times and the temporal expectancy window set by rhythmic sequences on a significant proportion of trials and confounds associated with timbre as a signal, the detection of which involves computationally complex multidimensional cues that are not likely ideal for detecting forward entrainment. Lin et al. do acknowledge some of these potential flaws, for example, use of short entraining sequences and averaging across expected cycles after termination of the entraining sequence. Acknowledging these, however, does not diminish their potentially detrimental impact. We have pointed out additional flaws and confounds that, collectively, may explain Lin et al.'s failure to detect forward entrainment in pitch discrimination, a finding that runs contrary to those from several other studies (Barnes & Johnston, 2010; Barnes & Jones, 2000; Ellis & Jones, 2010; Jones et al., 2002, 2006).

## CONFLICT OF INTEREST

The authors declare no conflict of interest.

## AUTHOR CONTRIBUTIONS

Kourosh Saberi and Gregory Hickok wrote the manuscript and performed the data analysis.

### PEER REVIEW

The peer review history for this article is available at https://publons.com/publon/10.1111/ejn.15778.

## Supporting information


**Figure S1.** Top panels show the time waveform of 40‐ms linear FM up and down sweeps. Bottom panels show that while their amplitude spectra are the same, their phase spectra are different.
**Figure S2.** Same as Figure 2 for a narrower sweep bandwidth (from 600 to 900 Hz). Note that while the curvature (second derivative) of the autocorrelation function shown in the middle panel changes signs, no peak is observed other than at zero lag, resulting in no pitch estimation at the model's output. The changing curvatures, however, may contribute to a dynamic timbre cue (see also **video 2** in the “Media Files” section of the online version).

**Video S1 and S2.** Video 1 shows the output of a running autocorrelation model of pitch extraction with a 5 ms exponential decay in response to a 40‐ms FM sound that linearly swept up from 500 to 2000 Hz. Top panel shows normalized autocorrelation amplitude as a function of autocorrelation lag. No pitch estimate is generated by the model (i.e., no secondary peak in the autocorrelation function). The bottom panel shows the response of a GammaTone filterbank to the same sound. The filterbank comprised 50 logarithmically spaced filters whose CFs ranged from 300 to ~3,000 Hz. The vertical red line shows current time.

**Video S2.** Video 2 shows the same as video 1 but for a narrower sweep bandwidth from 600 to 900 Hz.

**Audio S1.** Up sweep followed by down sweep at high frequencies: 40‐ms linear FM sweep from 5.5 to 7 kHz (and vice versa). Note that on each trial of Lin et al's experiment, only a single sweep was presented. Subjects had to judge the direction of the single sweep. The audio sample sequentially plays both types of up and down sweeps for comparison.

**Audio S2.** Same as audio demo 1 except for a 10‐ms FM that linearly swept from 0.5 to 2 kHz. Up sweep followed by down sweep.

**Audio S3.** Same as audio demo 1 except for a 40‐ms FM that linearly swept from 0.5 to 2 kHz. Up sweep followed by down sweep.

**Audio S4.** Same as audio demo 1 except for a 40‐ms FM that linearly swept from 0.6 to 0.9 kHz. Up sweep followed by down sweep.

## Data Availability

Matlab programs and data used to generate all figures in this paper are available at UCI's Data Repository (DRYAD). DOI: 10.7280/D19H6J.

## Footnotes

^1^
Lin et al.'s data and programs have been made publically available per EJN's requirements (https://osf.io/spt24/). However, we could not properly evaluate their analyses as the data and associated programs are not in a usable format. The uploaded folder contains over 1000 files with no instructions (or readme files) and no useful organization or file‐naming protocol. In addition, the program files fail to run as they are dependent on supplemental folders, programs or paths that either do not exist or cannot be properly accessed by their own programs.

^2^
Only the standard and comparison tones are shown, with the interleaving tones left out for clarity as they do not affect the model's pitch estimation for the standard and comparison.

^3^
Neither Lin et al. (2021) nor Wilsch et al. (2020) provide sufficient information about the bandwidth of the FM sweep used other than to say that it was set to generate performance in the range of 65–85% correct. Here, we have used a reasonable sweep extent of 1.5 kHz (0.5–2 kHz), though a lesser or wider bandwidth has no noticeable effect on the current analysis. This can be seen in Figure S2 and Video S2, which show the model's output for an FM sound that sweeps from 600 to 900 Hz. This latter range matches that used by Luo et al. (2007) in measuring psychophysical thresholds for brief FM pulses.
