# Supplementary material for: A critical analysis of Lin et al.'s (2021) failure to observe forward entrainment in pitch discrimination
Source: Eur J Neurosci. 2022 Aug 3;56(8):5191–200. doi: 10.1111/ejn.15778 (PMC9804316; doi:10.1111/ejn.15778)
Supplement: Supplementary file 1 — Figure S1. Top panels show the time waveform of 40‐ms linear FM up and down sweeps. Bottom panels show that while their amplitude spectra are the same, their phase spectra are different. Figure S2. Same as Figure 2 for a narrower sweep bandwidth (from 600 to 900 Hz). Note that while the curvature (second derivative) of the autocorrelation function shown in the middle panel changes signs, no peak is observed other than at zero lag, resulting in no pitch estimation at the model's output. The changing curvatures, however, may contribute to a dynamic timbre cue (see also video 2 in the “Media Files” section of the online version). [file EJN-56-5191-s002.pdf]

Supplemental Figures, Videos, and Audio Files to:
A critical analysis of Lin et al.'s (2021) failure to observe forward entrainment in pitch discrimination.

Authors: Kourosh Saberi and Gregory Hickok

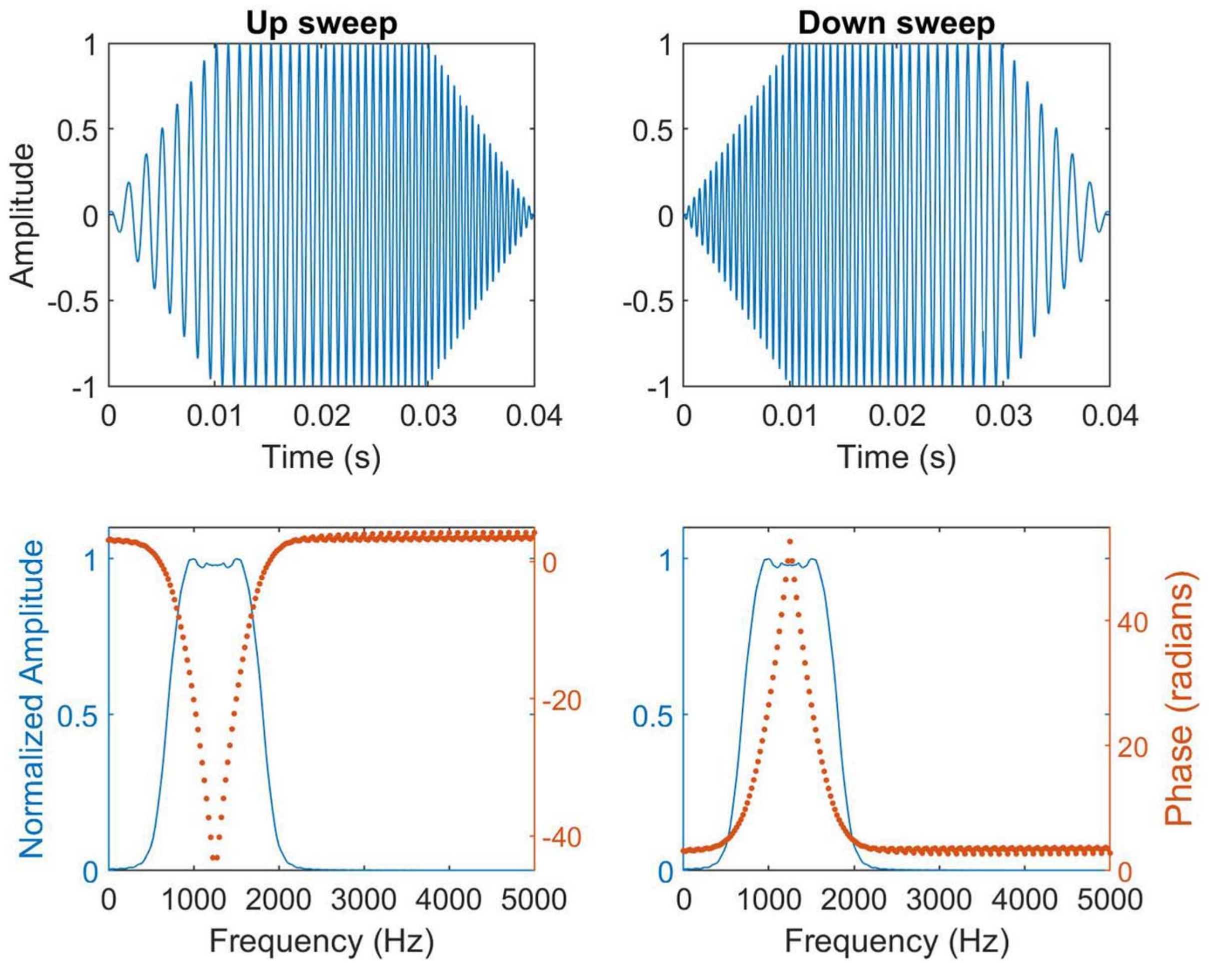

Supplemental Figure 1. Top panels show the time waveform of 40-ms linear FM up and down sweeps. Bottom panels show that while their amplitude spectra are the same, their phase spectra are different.

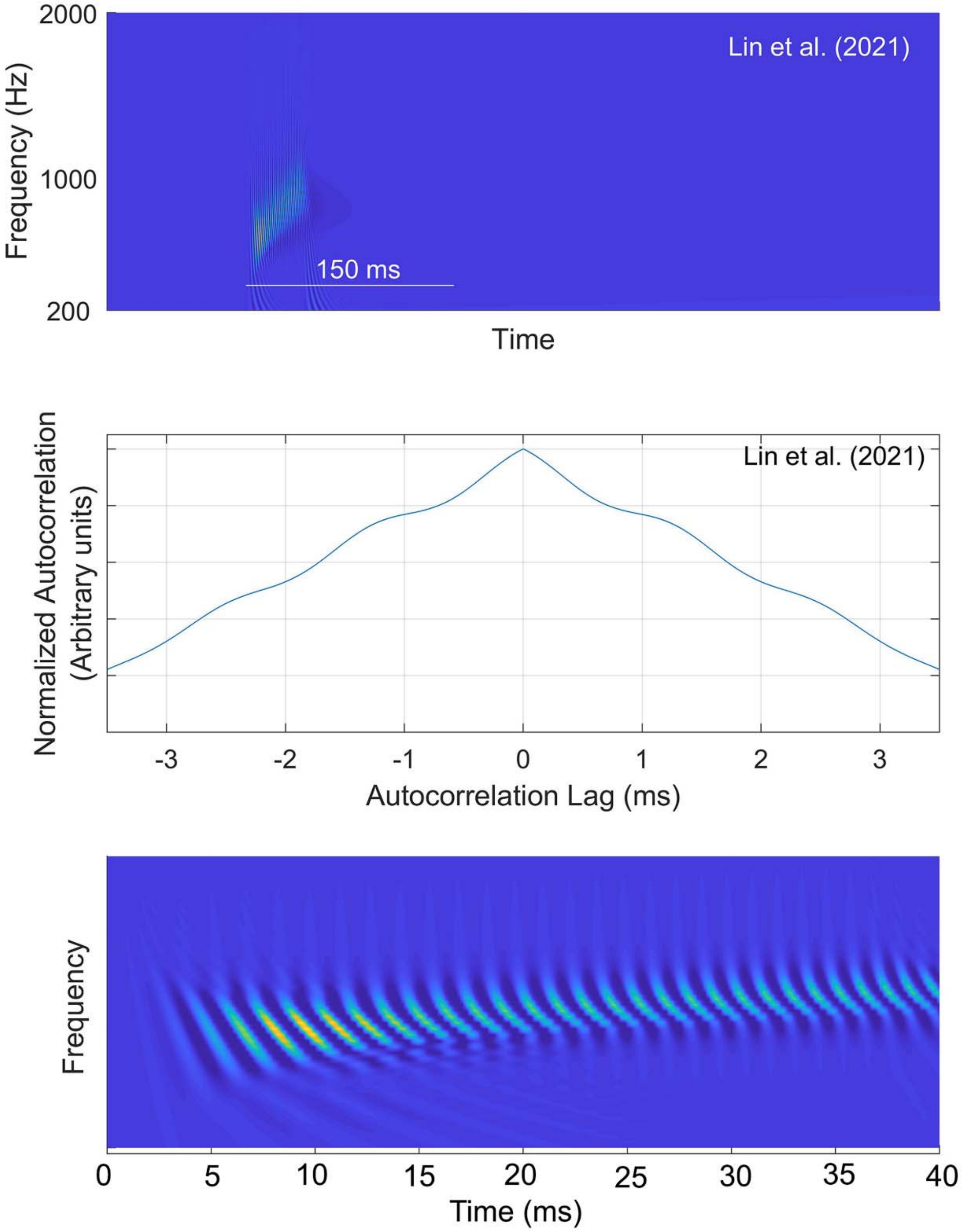

Supplemental Figure 2. Same as Fig. 2 for a narrower sweep bandwidth (from 600 to 900 Hz). Note that while the curvature (second derivative) of the autocorrelation function shown in the middle panel changes signs, no peak is observed other than at zero lag, resulting in no pitch estimation at the model's output. The changing curvatures, however, may contribute to a dynamic timbre cue (see also **video 2** in the "Media Files" section of the online version).

Audio and video demos are available in the “Media Files” section of the online version of this paper.

Video demos 1 and 2. Video 1 shows the output of a running autocorrelation model of pitch extraction with a 5ms exponential decay in response to a 40-ms FM sound that linearly swept up from 500 to 2000 Hz. Top panel shows normalized autocorrelation amplitude as a function of autocorrelation lag. No pitch estimate is generated by the model (i.e., no secondary peak in the autocorrelation function). The bottom panel shows the response of a GammaTone filterbank to the same sound. The filterbank comprised 50 logarithmically spaced filters whose CFs ranged from 300 to ~3000 Hz. The vertical red line shows current time. Video 2 shows the same as video 1 but for a narrower sweep bandwidth from 600 to 900 Hz.

Audio demo 1. Up sweep followed by down sweep at high frequencies: 40-ms linear FM sweep from 5.5 to 7 kHz (and vice versa). Note that on each trial of Lin et al’s experiment, only a single sweep was presented. Subjects had to judge the direction of the single sweep. The audio sample sequentially plays both types of up and down sweeps for comparison.

Audio demo 2. Same as audio demo 1 except for a 10-ms FM that linearly swept from 0.5 to 2 kHz. Up sweep followed by down sweep.

Audio demo 3. Same as audio demo 1 except for a 40-ms FM that linearly swept from 0.5 to 2 kHz. Up sweep followed by down sweep.

Audio demo 4. Same as audio demo 1 except for a 40-ms FM that linearly swept from 0.6 to 0.9 kHz. Up sweep followed by down sweep.
